# Excimer laser coronary angioplasty without stenting in a 37-year-old man with acute coronary syndrome involving left main trunk: a case report

**DOI:** 10.1093/ehjcr/ytae206

**Published:** 2024-04-18

**Authors:** Ryo Shigeno, Atsushi Hirohata

**Affiliations:** Department of Cardiovascular Medicine, The Sakakibara Heart Institute of Okayama, 2-5-1, Nakai-cho, Okayama, 700-0804, Japan; Department of Cardiovascular Medicine, The Sakakibara Heart Institute of Okayama, 2-5-1, Nakai-cho, Okayama, 700-0804, Japan

**Keywords:** Excimer laser coronary angioplasty, Acute coronary syndrome, Left main coronary artery, Left main trunk, Case report

## Abstract

**Background:**

Excimer laser coronary angioplasty (ELCA) is utilized to reduce thrombus in acute coronary syndrome (ACS). However, the feasibility and safety of ELCA for patients with ACS involving the left main trunk (LMT) and bifurcation, as well as the safety of a stentless strategy with ELCA, are not well-documented.

**Case summary:**

A 37-year-old man without any past medical history presented with chest pain. Electrocardiogram showed ST-segment elevation in leads I, aVL, and V2–V6. Emergent coronary angiography (CAG) showed a 99% stenosis from LMT to proximal left anterior descending artery (LAD). Intra-aortic balloon pumping (IABP) was initiated. Intravascular ultrasound revealed massive thrombus at the culprit lesion. Thrombus aspiration was not enough to reduce the thrombus, thus, we conducted thrombus vaporization with a 0.9 mm ELCA catheter. Coronary angiography after the procedure showed reduced thrombus with thrombolysis in myocardial infarction grade 3 flow. Considering his age and the complexity of stenting the LMT, we completed the procedure without stenting. After the intervention, we initiated triple antithrombotic therapy. On Day 3, we removed the IABP. On Day 11, CAG showed no significant stenosis. Optical coherence tomography revealed ulceration, indicating the presence of plaque disruption at the proximal LAD as the likely cause of thrombosis. With improvement in CAG findings, we stopped heparin and continued dual antiplatelet therapy. He was discharged on Day 20.

**Discussion:**

Excimer laser coronary angioplasty without stenting can be an option for the patients with ACS involving LMT, especially for younger patients who are suitable to avoid stenting on bifurcation lesions for lifelong management.

**Learning points**

- Excimer laser coronary angioplasty can be considered for lesions on the left main trunk to avoid bifurcation stenting, especially in young patients.
- Excimer laser coronary angioplasty without stenting can be considered even for the acute coronary syndrome caused by plaque rupture on the left main trunk and bifurcation.

## Introduction

Excimer laser coronary angioplasty (ELCA) is reported to vaporize thrombus and reduce the risk of distal embolization.^1^ A previous registry reported that ELCA was utilized in 53.4% of the patients in a real-world Japanese population,^2^ and another study revealed the feasibility and safety of ELCA for the treatment of patients with ST-segment elevation myocardial infarction.^3^ However, these studies did not specifically describe the data related to left main coronary artery disease. Moreover, while most of the patients who underwent ELCA had stents during the procedures,^2,3^ the optimal strategy regarding stenting after ELCA remains uncertain. Recently, there has been a universal tendency in world intervention strategies to return to the era of metal-free interventions. For example, the use of drug-coated balloons for small-vessel disease and side branches of bifurcations, as well as directional atherectomy, is observed as a stentless strategy.^4,5,6^ However, the feasibility and safety of ELCA for patients with acute coronary syndrome (ACS) involving the left main trunk (LMT) and bifurcation, as well as the safety of a stentless strategy with ELCA, are not well-documented.

We report a case of a young adult with ACS involving LMT who was treated with a stentless strategy with ELCA.

## Summary figure

**Table:** 

Time	Events
Day 1	The patient presented with chest pain.
	Electrocardiogram showed ST-segment elevation in leads I, aVL, and V2–V6.
	Coronary angiography (CAG) showed a 99% stenosis from left main trunk (LMT) to proximal left anterior descending artery (LAD).
	We completed percutaneous coronary intervention using excimer laser coronary angioplasty without stenting.
	Intra-aortic balloon pumping (IABP) was initiated with triple antithrombotic therapy.
Day 3	IABP was removed.
Day 11	CAG showed no significant stenosis in the LMT and LAD. We stopped heparin and continued dual antiplatelet therapy.
Day 20	He was discharged.

## Case summary

**Figure 1:**
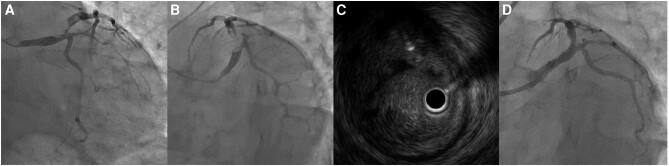
In coronary angiography at presentation, AP caudal (*A*) and LAO caudal (*B*) views showed a lesion from left main trunk to left anterior descending artery. Intravascular ultrasound showed massive thrombus (*C*). LAO caudal view after excimer laser coronary angioplasty revealed improvement of coronary stenosis (*D*). AP, anteroposterior; LAO, left anterior oblique.

**Figure 2:**
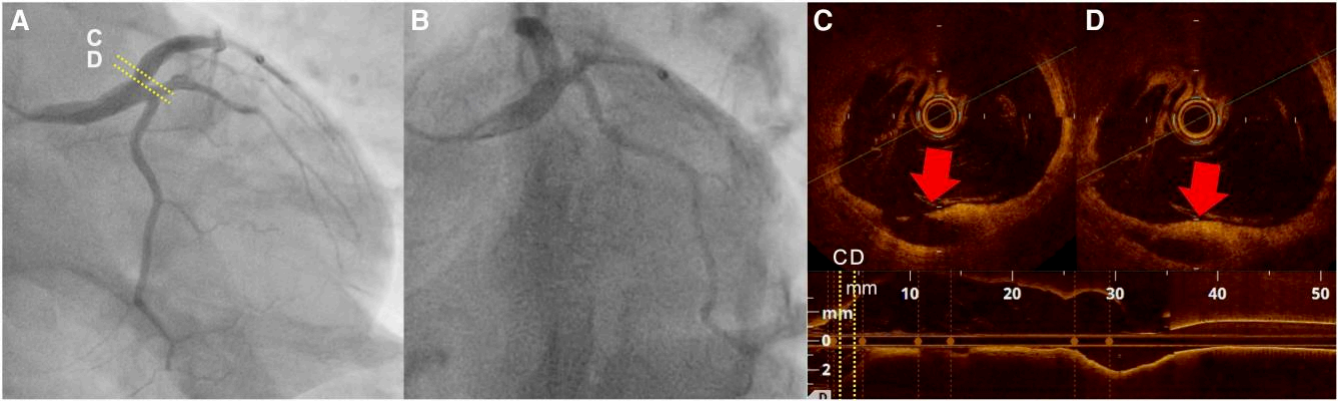
In coronary angiography on Day 11, AP caudal (*A*) and LAO caudal (*B*) views showed no significant stenosis. Optical coherent tomography revealed ulceration (red arrow) at the proximal left anterior descending artery (*C* and *D*). AP, anteroposterior; LAO, left anterior oblique.

A 37-year-old man without any past medical history presented with squeezing chest pain, accompanied by cold sweats. On arrival, his blood pressure was 127/87 mmHg, pulse rate was 96 beats/min, respiratory rate was 25 breaths/min, body temperature was 35.7°C, and SpO_2_ was 97% (room air). Heart and pulmonary sounds were not significant. Electrocardiogram (ECG) showed ST-segment elevation in leads I, aVL, and V2–V6. Transthoracic echocardiography mainly showed akinesis from the anteroseptal wall to the anterolateral wall of the left ventricle (see Supplementary material online, *Video S1*). With a presumed diagnosis of ACS, emergent coronary angiography (CAG) was performed, which showed a 99% stenosis from LMT to proximal left anterior descending artery (LAD) (*Figure 1A* and *B*, Supplementary material online, *Videos S2* and *S3*). We decided to perform percutaneous coronary intervention (PCI) and prescribed 200 mg of acetylsalicylic acid and 20 mg of prasugrel. While the patient’s vital signs were normal, considering the severe lesion, intra-aortic balloon pumping (IABP) was initiated. Intravascular ultrasound revealed massive thrombus and attenuated plaque, indicating the presence of lipid-rich burden on the culprit lesion (*Figure 1C*). Thrombus aspiration using 7Fr Rebirth Pro2 (NIPRO, Osaka, Japan) was not enough to reduce the thrombus, thus, we conducted thrombus vaporization with ELCA catheter. The 0.9 mm catheter size was selected for its advantage allowing longer laser emissions at once. The fluence and frequency were set at 80 mJ/mm^2^ and 80 Hz. We completed eight sessions, each lasting 10 s, with slow advancement of the catheter at 0.5 mm/s to acquire a larger lumen. Continuous saline infusion at a rate of 2–3 mL/s was maintained to prevent heat build-up caused by laser energy. After ELCA, balloon dilation was performed using Regnam 4.0–15 mm (NIPRO, Osaka, Japan). Coronary angiography after the procedure showed reduced thrombus with thrombolysis in myocardial infarction grade 3 flow (*Figure 1D*). Considering his age and the complexity of stent implantation on the LMT, we completed the procedure without stenting. After the intervention, we initiated triple antithrombotic therapy: intravenous unfractionated heparin, 100 mg of acetylsalicylic acid, and 3.75 mg of prasugrel. Twelve hours after the PCI, peak creatine kinase was observed at 4356 U/L (reference range: 59–248 U/L). On Day 3, ECG showed ST-segment resolution and no chest symptoms were observed, thus, we removed the IABP. To prevent acute occlusion of coronary artery, we maintained him on triple antithrombotic therapy after the removal of IABP. On Day 11, CAG showed no significant stenosis in the LMT and LAD (*Figure 2A* and *B* and Supplementary material online, *Video S4*). Optical coherence tomography revealed ulceration, indicating the presence of plaque disruption at the proximal LAD as the likely cause of thrombosis (*Figure 2C* and *D*). With improvement in CAG findings, we stopped heparin and continued dual antiplatelet therapy (DAPT). After rehabilitation, he was discharged on Day 20. At discharge, a three-month course of DAPT, which is relatively short-term for ACS after PCI, was planned. This decision was made because the risk of coronary thrombosis after de-escalating to single antiplatelet therapy was estimated to be low, given the absence of stenting in this case. Three months after the discharge, he showed no anginal symptoms. Therefore, we stopped acetylsalicylic acid and continued 3.75 mg of prasugrel combined with rosuvastatin, enalapril, and bisoprolol.

## Discussion

The feasibility and safety of ELCA for patients with ACS involving the LMT and bifurcation without stenting are not well-investigated, and this is an important issue particularly for young adults who are suitable to avoid stenting on LMT for lifelong management. We report a case of a young adult with ACS involving LMT who was treated with a stentless strategy with ELCA.

The action of ELCA is based on its photochemical (fracture of molecular bonds), photokinetic (tissue vaporization), and photothermal effects (clearance of by-products), which enable its application in ACS.^7^ Previous case series demonstrated the feasibility of ELCA for ACS caused by unprotected left main coronary artery, which was defined by angiographically significant stenosis of the LMT without patent surgical grafts to the left coronary artery system.^8^ In this report, all cases completed the procedure and received stent implantation, which suggested feasibility of ELCA for ACS involving LMT. Concerning the stentless strategy, a previous report documented a case of ACS with a left main bifurcation lesion treated with ELCA without stenting.^9^ The lesion of this case, as assessed by optical coherence tomography, was neither plaque rupture nor calcified nodule. A recent study showed the feasibility of antiplatelet therapy alone for the patients with ACS caused by plaque erosion defined by optical coherence tomography.^10^ However, the safety of a stentless strategy for the lesions derived by coronary plaque rupture remains unknown. In the current case, although a plaque rupture was suspected as the primary cause of thrombosis, the patient was successfully treated with ELCA without stenting. A recent study showed that 24 out of 86 patients with ACS treated with a drug-coated balloon and ELCA underwent unplanned stenting due to complications such as coronary dissection, residual thrombus, recoiling, failed laser catheter-delivery, slow-flow after laser application, and haemodynamic instability.^11^ Therefore, selected patients without these findings might be candidates for ELCA without stenting.

In terms of antithrombotic therapy after PCI, we selected triple antithrombotic therapy that included intravenous unfractionated heparin, acetylsalicylic acid, and prasugrel. After ELCA without stenting, a prior case selected dual antiplatelet therapy and another case adopted anticoagulation therapy alone.^9,12^ The optimal antithrombotic therapy following ELCA without stenting remains uncertain. Our case suggested that patients with massive thrombus and without high bleeding risks might be potential candidates for short-term triple antithrombotic therapy.

In the current guideline, patients with ST-segment elevation myocardial infarction are recommended to undergo PCI if the procedure is feasible and implant stents in the infarct-related artery during the index PCI.^13^ A recent study reported that while PCI for ostial or shaft lesions of LMT was not associated with a higher rate of mortality compared with coronary artery bypass grafting, PCI for LMT bifurcation lesions had a higher rate of mortality and target vessel revascularization in the long-term.^14^ Moreover, another study demonstrated that young patients (<45 years) who underwent PCI for LMT and/or three-vessel disease had a higher rate of major adverse cardiac and cerebrovascular events and repeat revascularization.^15^ In the current case, a young adult with ACS involving the LMT was successfully treated using a stentless strategy with ELCA. This approach may be especially beneficial for younger patients to avoid adverse long-term outcomes related to stent implantation on LMT bifurcation lesions.

## Conclusions

In conclusion, ELCA without stenting can be a potential therapeutic option for the patients with ACS involving LMT, especially for younger patients who are suitable to avoid stenting on bifurcation lesions.

## Supplementary Material

ytae206_Supplementary_Data

## Data Availability

The data underlying this article will be shared on reasonable request to the corresponding author.

## Lead author biography

Ryo Shigeno graduated from Shimane University in Japan. He is interested in structural heart disease intervention and heart failure.

## Supplementary material

**Consent:** The authors confirm that written consent for submission and publication of this case report, including image(s) and associated text, has been obtained from the patient in line with COPE guidance.

**Funding:** None declared.
